# Computational Analysis of the Influence of Menopause and Ageing on Bone Mineral Density, Exploring the Impact of Bone Turnover and Focal Bone Balance—A Study on Overload and Underload Scenarios

**DOI:** 10.3390/life13112155

**Published:** 2023-11-02

**Authors:** Feliciano Franco, Carlos Borau Zamora, Diego Martín Campana, Marcelo Eduardo Berli

**Affiliations:** 1 Instituto de Bioingeniería y Bioinformática, Universidad Nacional de Entre Ríos, Consejo Nacional de Investigaciones Científicas y Técnicas, Ruta 11, Km 10, Oro Verde 3100, Argentina; diego.campana@uner.edu.ar; 2 Facultad de Ingeniería, Universidad Nacional de Entre Ríos, Ruta 11, Km 10, Oro Verde 3100, Argentina; marcelo.berli@uner.edu.ar; 3 Multiscale in Mechanical and Biological Engineering, Department of Mechanical Engineering, University of Zaragoza, 50018 Zaragoza, Spain; cborau@unizar.es; 4 Centro Universitario de la Defensa de Zaragoza, 50090 Zaragoza, Spain

**Keywords:** menopause, bone remodeling, computational simulation, mathematical model, osteoporosis, bone health

## Abstract

This study aims to investigate the impact of hormonal imbalances during menopause, compounded by the natural ageing process, on bone health. Specifically, it examines the effects of increased bone turnover and focal bone balance on bone mass. A three-dimensional computational bone remodeling model was employed to simulate the response of the femur to habitual loads over a 19-year period, spanning premenopause, menopause, and postmenopause. The model was calibrated using experimental bone mineral density data from the literature to ensure accurate simulations. The study reveals that individual alterations in bone turnover or focal bone balance do not fully account for the observed experimental outcomes. Instead, simultaneous changes in both factors provide a more comprehensive explanation, leading to increased porosity while maintaining the material-to-apparent density ratio. Additionally, different load scenarios were tested, demonstrating that reaching the clinical osteoporosis threshold is independent of the timing of load changes. However, underload scenarios resulted in the threshold being reached approximately 6 years earlier than overload scenarios. These findings hold significant implications for strategies aimed at delaying the onset of osteoporosis and minimizing fracture risks through targeted mechanical stimulation during the early stages of menopause.

## 1. Introduction

Bone is a dynamic tissue subject to continuous remodeling orchestrated by specialized cellular units known as basic multicellular units (BMUs) in a sequence of well-coordinated metabolic processes [1,2]. This delicate balance is overseen by osteoclasts, osteoblasts, and various other cell types, such as osteocytes, bone lining cells, osteal macrophages, and vascular endothelial cells, all functioning within the microenvironment of the BMU [3,4]. These processes can be broadly categorized into two main functions: resorption and formation. It is the precise interplay between these functions that ensures the bone’s microstructure aligns with its functional demands [2,5].

While, in young individuals, bone turnover through remodeling is advantageous, various natural and pathological factors can disrupt this delicate balance, resulting in net bone alterations that can, in certain cases, be detrimental. One of the most well-studied factors in this regard is menopause, which holds significant implications for women experiencing this physiological transition [6]. The onset of menopause, marked by the cessation of menstrual cycles due to declining ovarian follicular activity, leads to lowered serum estrogen levels and disrupts the usual bone remodeling process [2,7]. This disruption primarily impacts two factors crucial to Basic Multicellular Units (BMUs): the activation frequency, closely related with the bone turnover, and the focal bone balance. The cellular intricacies of bone turnover and focal bone balance have been explored in earlier studies [8,9]. As a consequence of the loss of hormonal anti-catabolic action, the frequency of BMU activation increases from premenopausal levels and the remodeling balance tends toward net resorption, resulting in an excessive loss of bone density [10,11]. Also, age-related effects such as decreased intestinal calcium absorption [12], vitamin D deficiency [13], and impaired synthesis of active 1,25-dihydroxyvitamin D3 by the kidneys [14] contribute to accelerated bone resorption [15,16]. Conversely, retirement frequently leads to a decrease in the mechanical stimulation of bones. In addition, due to the absence of down-regulation in estrogen-mediated sclerostin production by osteocytes, there is also a reduction in osteoid formation [7,10]. The resulting imbalance between resorption and formation alters bone quantity and quality. As a result, the material performance, i.e., strength and fragility, are affected [17]. Understanding the influence of menopause process and its different stages on bone health can help individuals attend this significant life transition.

The mathematical modeling of bone remodeling in peri- and postmenopausal stages allows for a more detailed description of the process of bone density loss during these stages and the development of osteoporosis [18]. In this context, studies have utilized phenomenological models based on the work by Hernández et al. [19]. Such models employ variables averaged over the bone volume to evaluate elastic parameters based on bone volume fraction and mineralization content. These variables undergo changes corresponding to the density of activated BMUs at a given time. The activation process is mediated by the stimulus level, a scalar variable proportional to deformations at each point, thereby establishing a feedback system [2,20].

The impact of maximum bone density achieved throughout life, age of menopausal onset, and the rate of age-related bone loss on the development of postmenopausal osteoporosis (PMO) has been characterized [21]. Temporal and permanent dynamic changes in model parameters, including BMU activation rate and the degree of imbalance between resorption and formation, have been calibrated against experimental values, both individually and in combination, to reflect the evolution of peri- and postmenopausal BMD [22]. The work by Hernández et al. [22] is limited to 9.5 years, including premenopause and menopause, during whichconclusions were drawn by aligning the computational results with experimental data. While excellent agreement between these results was achieved, the effects of model parameters could be influenced by the BMD decreasing after menopause, an extension for which the experimental results they considered do not account. In a later work, Hernándezd et al. [21] proposes a model in which the focal balance has a sustained and constant modification after menopause, showing that this generates a constant loss of BMD that is attributed to ageing. However, although his conclusions are highly valuable for clarifying the factors that lead to osteoporosis, this model was not tested against experimental data throughout the complete simulation period. Recently, Martínez Reina et al. [23] proposed a model of bone remodelling incorporating the effect of bone mineralization, micro-damage, and mechanobiological feedback. They investigated a variety of treatment scenarios, with an emphasis on the combined effects of mechanical loading (including overuse and disuse) together with denosumab treatment in PMO. Their findings include that late treatment for PMO patients shows higher local failure risk compared to early treatment. They showed that an increment of exercise is followed by a decrease in the risk of failure. Conversely, a decrease in mechanical loading reduces the effectiveness of the treatment. and this could seriously compromise bone integrity. The timing of denosumab treatment initiation plays a critical role. A delayed start may potentially increase the risk of failures, especially in cases where there is a significant increase in load [23].

Previous computational works predict BMD evolution in a representative volume of a human bone cross-section, neglecting the potential effects of macroscopic geometry and stress distribution at the organ level. It is, however, important to investigate whether a whole bone has the same loss, considering that different parts of its geometry are subjected to different loads and contain different degrees of mineralization as starting conditions. Therefore, a more comprehensive approach is necessary to determine whether the phenomena observed in previous studies are specific to particular regions or affect the entire bone uniformly. In this sense, ref. [24] conducted dual-energy X-ray absorptiometry (DXA) measurements of BMD at posteroanterior lumbar spine and proximal femur in female subjects of age 20–89 years from different regions of China. Results for the total femur showed a steady decline in BMD after the age of 40 years, reaching an almost 36% reduction at 80 years compared to the maximum value reached during the period of 30–35 years. Indeed, Bonicelli et al. [25] show that maturity is reached at 35 years exactly, and afterwards bone deteriorates at the bone tissue level. A computational model capable of simulating this behaviour for realistic geometries and loads would provide a valuable tool for predicting the evolution of bone mass in women in clinical situations of interest. Recent studies have pursued this goal to obtain a better understanding of the resorption process [8] and achieved validation in three-dimensional models [9], albeit without incorporating menopausal effects. Another aspect not considered in conjunction with these effects is the self-accommodation of bone tissue to the stimuli it undergoes [26]. The objective of the present work is to analyze these effects in a three-dimensional model of the femur under realistic loading conditions, with a particular focus on the biological implications of the model results.

## 2. Materials and Methods

The proposed model is based on previous works [8,9]. In this section, we compile a summary of the most relevant equations to understand the model. We also include new equations corresponding to menopause and agening, which were not present in previous versions of the model. Further details can be found in the aforementioned articles.

### 2.1. Tissue Composition

Within the bone, two distinct phases are present: the bone tissue itself and the pores. The bone tissue comprises a mineral component, an organic component, and water. This concept is summarized by Equation (1):

(1) $$\begin{array}{cc} V_{t} & =V_{b}\left(t\right)+V_{p}\left(t\right)\\ \; & =V_{m}\left(t\right)+V_{o}\left(t\right)+V_{w}\left(t\right)+V_{p}\left(t\right), \end{array}$$

where $V_{b}\left(t\right)$ is bone tissue volume, $V_{p}\left(t\right)$ is the pore volume, $V_{m}\left(t\right)$ is the mineral volume, $V_{o}\left(t\right)$ is the organic volume and $V_{w}\left(t\right)$ is the water volume. To compute these variables, they are normalized according to the following equations:

(2) $$\begin{array}{ccc} v_{b}\left(t\right)=\frac{V_{b}\left(t\right)}{V_{t}} & \; & v_{m}\left(t\right)=\frac{V_{m}\left(t\right)}{V_{b}\left(t\right)}\\ v_{o}\left(t\right)=\frac{V_{o}\left(t\right)}{V_{b}\left(t\right)} & \; & v_{w}\left(t\right)=\frac{V_{w}\left(t\right)}{V_{b}\left(t\right)} \end{array}$$

Organic content is assumed to be constant at $v_{o}=3/7$ [27]. As a consequence of Equation (2), it follows that:

(3) $$v_{m}\left(t\right)+v_{o}+v_{w}\left(t\right)=1$$

Therefore, the model describes the tissue composition using only the variables $v_{b}\left(t\right)$ and $v_{m}\left(t\right)$, as the mineral volume replaces the water volume. It is convenient to quantify the mineral content by the ash fraction α, defined as the ratio of the mineral mass to the dry mass of the tissue:

(4) $$\alpha =\frac{\rho_{m}v_{m}}{\rho_{m}v_{m}+\rho_{o}v_{o}},$$

where $\rho_{m}=3.2\;g/{\mathrm{cm}}^{3}$ is the mineral content density and $\rho_{o}=1.1\;g/{\mathrm{cm}}^{3}$ is the organic content density [27]. The elastic modulus can be estimated from the variables $v_{b}$ and α. This relationship for this isotropic model is explored by Hernández et al. [28]:

(5) $$E=84370\;v_{b}^{2.58}\alpha^{2.74}\;\mathrm{MPa}$$

Experimentally, bone mineral density (BMD) is obtained by bone DXA tests, as this is an indicator of bone health and strength; the early detection of low values can allow for interventions to help prevent or slow down the progression of osteoporosis [29]. In our model, Equation (6) estimates the value of BMD as a function of $v_{b}$ and $v_{m}$.

(6) $$\mathrm{BMD}=\rho_{m}v_{m}v_{b}$$

Other important quantities are material density $\rho_{\mathrm{mat}}$ and apparent density $\rho_{\mathrm{app}}$. The former represents the mass of bone tissue per unit of bone volume (excluding pore volume) and is related to the mineral content. The latter refers to the mass of bone tissue per unit of total tissue volume (including pore volume), which is more related to bone volume fraction and porosity. These quantities are interconnected in a specific way in real bones, as demonstrated in the work by Zioupos et al. [30]. This relationship is crucial and was previously used to validate the results in other works [8,9]. Likewise, it is also used, in the same context, in the present study. These quantities can be computed from previous tissue variables as follows:

(7) $$\begin{array}{cc} \rho_{\mathrm{mat}} & =\rho_{m}v_{m}+\rho_{o}v_{o}+\rho_{w}v_{w}, \end{array}$$

(8) $$\begin{array}{cc} \rho_{\mathrm{app}} & =\rho_{\mathrm{mat}}v_{b}, \end{array}$$

where $\rho_{w}=1.0\;g/{\mathrm{cm}}^{3}$ is the aqueous content density [27].

### 2.2. Remodeling Dynamics

Based on previous works models [8,9], the rate of change of $v_{b}$ is determined by the difference between the rates of bone formation and bone resorption:

(9) $$\dot{v_{b}}\left(t\right)={\dot{v}}_{f}\left(t\right)-{\dot{v}}_{r}\left(t\right)$$

These rates are given by the following equations:

(10) $$\begin{array}{c} {\dot{v}}_{f}\left(t\right)=\frac{1}{T_{F}}\int_{t-T_{R}-T_{I}-T_{F}}^{t-T_{R}-T_{I}}N_{\mathrm{BMU}}\left(\tau \right)A_{\mathrm{BMU}}^{f}{\mathrm{bb}}^{(}\tau )v_{\mathrm{BMU}}d\tau \end{array}$$

(11) $$\begin{array}{c} {\dot{v}}_{r}\left(t\right)=\frac{1}{T_{R}}\int_{t-T_{R}}^{t}N_{\mathrm{BMU}}\left(\tau \right)A_{\mathrm{BMU}}^{v}\mathrm{BMU}d\tau \end{array}$$

Here, $T_{R}$, $T_{I}$ and $T_{F}$ represent the duration of the resorption, inversion and formation intervals undergone by the BMUs, respectively. $A_{\mathrm{BMU}}$ denotes the cross-sectional area of remodeling, $v_{\mathrm{BMU}}$ represents the rate of progression of the BMUs, $f_{\mathrm{bb}}$ stands for the focal bone balance, and $N_{\mathrm{BMU}}$ represents the number of active BMUs per unit volume. The rate of BMU activation depends on both the biological and mechanical-driven frequency of activation, denoted as $f_{\mathrm{or}}$, and the free surface area per unit of volume $S_{v}$:

(12) $$\frac{dN_{\mathrm{BMU}}}{dt}=f_{\mathrm{or}}S_{v}$$

The frequency of activation is inhibited by the mechanical stimulus and cannot exceed the biological frequency set by genetic and hormonal influences. This can be expressed mathematically as:

(13) $$f_{\mathrm{or}}=f_{\mathrm{bio}}^{\left(1-\frac{\zeta }{\zeta +\zeta_{\mathrm{ref}}}\right)}$$

Here, $f_{\mathrm{bio}}$ represents the biological frequency of activation, and ζ represents the mechanical stimulus, which will be discussed below. $\zeta_{\mathrm{ref}}$ is the reference mechanical stimulus, which represents the usual mechanical stimulus.

According to Hernández et al. [21], the activation frequency of the BMUs involved in the bone remodeling process is highly influenced by menopause. This activation frequency, denoted as $f_{\mathrm{bio}}$, is influenced by hormonal changes during menopause, which increase BMU activity due to a decrease in hormonal inhibition [10]. Hence, this increase in activity is reflected as an increase in the $f_{\mathrm{bio}}$ parameter.

Remodeling occurs over the tissue’s free surface area; therefore, tissue formation develops in layers, where the superficial layers are younger and less mineralized than the deeper ones [9]. During bone remodeling, osteoclasts are the initial cells that act on the bone surface, removing old bone in the areas where new non-mineralized bone (osteoid) will be deposited by osteoblasts. Due to the inherent nature of the remodeling process, at moderate levels of remodeling activity, resorption primarily affects only the superficial and less mineralized tissue. However, as remodeling activity increases, particularly during menopausal process, resorption extends to the innermost and more mineralized tissue. The increased activity of BMUs implies that more bone sites are being replaced, resulting in the transformation of bone into a younger and less mineralized tissue before the long mineralization period occurs [9]. This leads to a depletion not only in bone volume but also in its mineral content. The progressive involvement of deeper and denser bone regions contributes to the overall loss of bone mass and mineral content.

**Figure 1:**
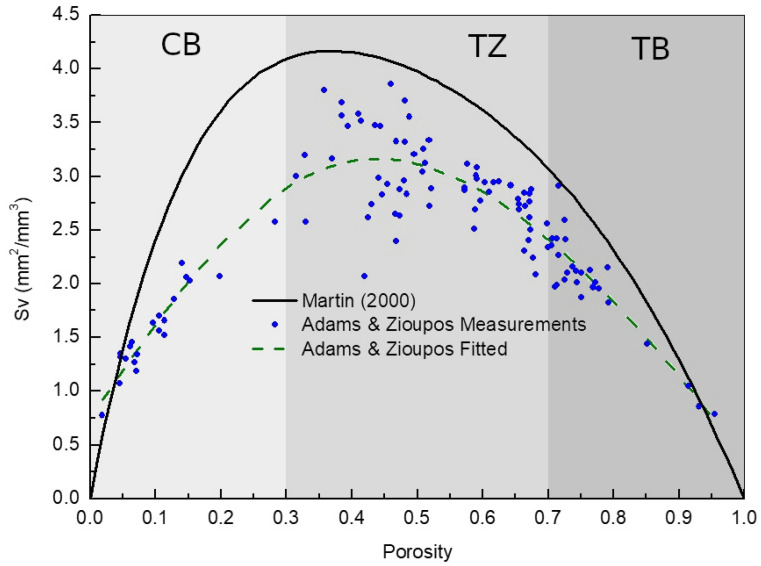
Free surface area $S_{v}$ as a function of porosity from the analysis produced by Martin [31] (solid curve) and precise measurements by Adams et al. [32] (circles with a dashed line, which is a fifth degree polynomial fit). Different shaded areas show the regions of cortical bone (CB), trabecular bone (TB) and transition zone (TZ) [8]. Figure adapted from Berli et al. [8].

The free surface area per unit of volume depends on the porosity of the tissue and has been characterized by Martin [31] and Adams et al. [32] (see Figure 1). As the number of active BMUs is limited by the free surface area, several levels of activity can coexist at the same time for the same mechanical stimulus but in different tissue localizations. Figure 1 shows that greater activity is expected in transitional tissue (medium-range porosity levels) than in cortical and trabecular bone (extremely low–high porosity levels). As a consequence, an increase in porosity in cortical bone leads to an increase in remodeling. In contrast, a decrease in porosity in trabecular bone leads to an increase in remodeling.

Another critical variable is the focal bone balance, denoted as $f_{\mathrm{bb}}$ (Equation (10)), which represents the inclination of BMUs towards bone formation or resorption. As an individual ages, bone resorption becomes the dominant process [33]. Under normal conditions, there is an equilibrium between osteoclastic bone resorption and osteoblastic bone deposition, which is maintained until around the age of 30 [34]. After this age, an imbalance occurs, favoring osteoclastic resorption and resulting in a sustained loss of bone mass throughout life [35]. This imbalance is not gender-specific but is more pronounced during the menopausal period for women, as highlighted by Khosla et al. [10].

**Figure 2:**
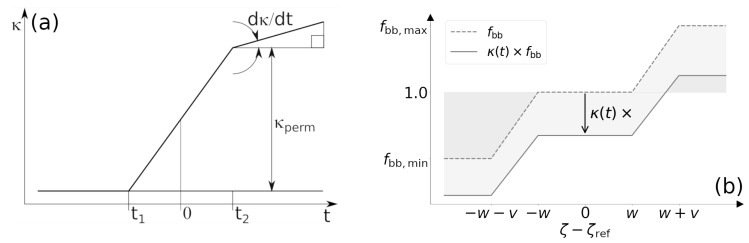
(**a**) κ(t) functions for parameter variation. These functions affects the parameters $f_{\mathrm{bio}}$ and $f_{\mathrm{bb}}$, gradually increasing the former while decreasing the latter over time. The beginning of the perimenopausal stage is depicted by $t_{1}$, and the beginning of the postmenopausal stage by $t_{2}$. The value 0 indicates the instant of the last menses. (**b**) κ effect on $f_{\mathrm{bb}}$ curve. As time progresses, as κ(t) decreases, the $f_{\mathrm{bb}}$ curve shifts downward, indicating a diminishing capacity for bone formation. The width of the dead zone is depicted by *w*, and the width depicts the linear variation zone by *v*.

By examining Equations (10) and (11), it can be observed that the difference in Equation (9) is directly proportional to $f_{\mathrm{bb}}$. The focal bone balance $f_{\mathrm{bb}}$ is a function of the difference between the daily mechanical stimulus ζ and the reference mechanical stimulus $\zeta_{\mathrm{ref}}$, expressed as $f_{\mathrm{bb}}=f_{\mathrm{bb}}\left(\zeta -\zeta_{\mathrm{ref}}\right)$. This dependency is depicted in Figure 2b. The daily mechanical stimulus, denoted as ζ, represents the daily deformation history and is dependent on the equivalent strain generated from the strain energy density at each point in the domain, as well as the number of load cycles applied, following the approach proposed by Mikić and Carter [36]:

(14) $$\zeta =\sqrt[{m}]{\sum_{i=0}^{n}N_{i}\varepsilon_{i}^{m}}$$

Here, $N_{i}$ is the number of cycles and $\varepsilon_{i}$ is the effective strain of the *i*-th load state, and m=4 is a weight parameter [36]. The reference mechanical stimulus is the typical level of mechanical stimuli that receptor cells detect under normal circumstances, and gradually adapts to the daily mechanical stimuli [37]. This behavior is referred to as accommodation and can be modeled by Equation (15), where the parameter ϕ represents the accommodation rate.

(15) $$\frac{d\zeta_{\mathrm{ref}}}{dt}=\phi \left(\zeta -\zeta_{\mathrm{ref}}\right)$$

All the parameters used for the bone remodeling model can be seen in Table 1.

### 2.3. Menopause Model

To model the effects of menopause on bone remodeling, Hernández et al. [22] incorporated time-dependent changes, independent of mechanical stimuli, to the parameters $f_{\mathrm{bb}}$ and $f_{\mathrm{bio}}$. In this study, these changes over time are implemented using multiplication functions, denoted as κ(t). These functions take into account not only the effects during menopause but also the ageing process that occurs after menopause. The shape of these functions is shown in Figure 2a. In that figure, t=0 represents the instant of the last menses; the values $t_{1}$ and $t_{2}$ are set at −4 years and +4 years, respectively, based on hormonal fluctuation onset and bone mass loss trends [22]. The value of $\kappa_{\mathrm{perm}}$ signifies the permanent change in the baseline of $f_{\mathrm{bio}}$ or $f_{\mathrm{bb}}$ due to the menopausal effect, while the long-term slope depicted in Figure 2a represents a gradual drift in these parameters due to the ageing effect. This approach differs from the work by Hernández et al. [21], which relates menopausal effects to an increase in bone turnover and ageing effects to an increase in bone resorption.

Hernández et al. [22] assume only permanent changes in the parameters that model the rate of bone remodeling activation ($f_{\mathrm{bio}}$) and the osteoblast/osteoclast focal balance ($f_{\mathrm{bb}}$), finding that both effects play important roles in the evolution of BMD. In fact, they showed that a permanent change in both parameters could explain the BMD trends by fitting the computational results to experimental data that are limited to the menopausal period. In the present work, however, it is proposed that, after the menopausal period, the mentioned parameters continue to vary due to the effects of ageing. The different hypotheses are modeled by assigning values to $\kappa_{\mathrm{perm}}$ and to long-term slope. In the case of the activation frequency ($f_{\mathrm{bio}}$), changes occur, increasing activity (κ(t)>1), while for the focal balance, the changes are decreasing (0<κ(t)<1), implying that there is a removal process that increasingly exceeds tissue deposition.

The impact of κ(t) on $f_{\mathrm{bb}}$ can be observed in Figure 2b. As κ is less than 1, its decrease shifts the $f_{\mathrm{bb}}$ curve downwards. In that case, the dead zone, which is the interval between *−w* and *w* where $\zeta -\zeta_{\mathrm{ref}}$ gradually converges to zero due to the accommodation effect (Equation (15)), exhibits values lower than 1. Consequently, there is a net resorption in the long term. Additionally, when κ becomes sufficiently small, no bone formation occurs regardless of the value of $\zeta -\zeta_{\mathrm{ref}}$. Under such conditions, mechanical stimulation does not lead to bone formation.

### 2.4. Mechanical Model

**Figure 3:**
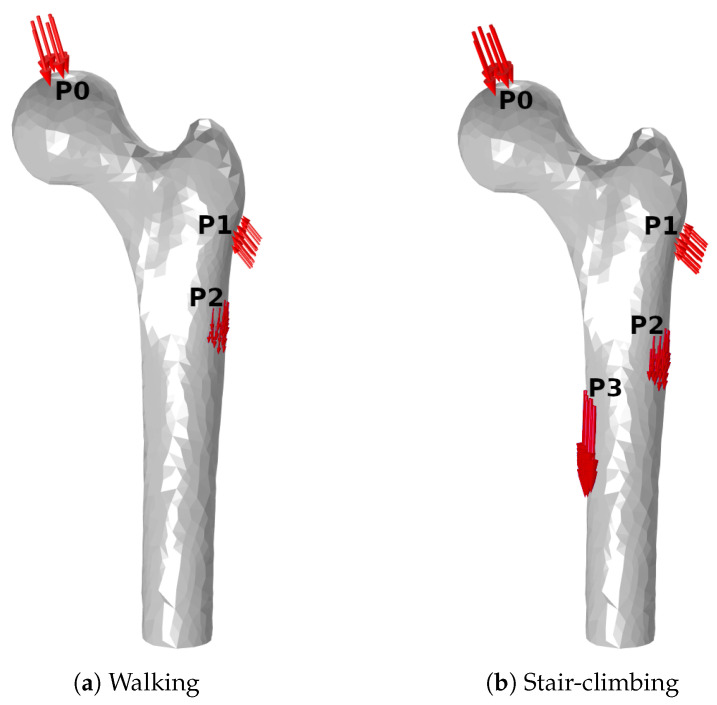
Diagram of loadings showing the application points and direction of the forces. The corresponding values are listed in Table 2.

The region of interest is limited to the proximal half of the femur, as shown in Figure 3. The applied loads correspond to walking and stair-climbing, and are statically applied, following the approach suggested in the research work by Martínez-Reina et al. [38]. The value of each applied load corresponds to the instant of maximum contact force for walking and the instant of maximum torsional moment for stair-climbing, which are used to estimate ζ. This approach only focuses on the most relevant muscles and considers that the subject performs 5000 steps per day in walking gait and 500 steps per day in stair-climbing. These forces and their application points were measured by Heller et al. [39]. Table 2 list these forces, and Figure 3 depict their application points.

Bone is assumed to be a linear and locally isotropic elastic material, with the Poisson’s ratio ν=0.3 [38]. The elastic modulus *E* is given by Equation (5). The displacement field is solved iteratively using the finite element method, with each iteration representing one simulated day. The algorithm updates material variables $v_{b}$ and α at each iteration. The bone model starts with an initial equilibrium density distribution for the imposed loads after reaching a stationary state, which was validated with experimental values by Berli et al. [9].

The mechanical model is implemented in COMSOL Multiphysics 5.3a software, which interacts iteratively with the remodeling model implemented in Java. The software was executed using the available resources at the Advanced Computing Laboratory, Faculty of Engineering, National University of Entre Ríos, to ensure efficient model resolution. The HPC cluster system consists of 12 nodes with Intel Xeon E5-2670 v3 2.5 GHz processors, each equipped with 128 GB RAM running at 2133 MT/s. These nodes are interconnected using an Infiniband SX6012F switch. As a reference, simulating 20 years of bone evolution using the model takes approximately 38.5 h of computational time when it is allocated on one node (24 cores). Prior to starting the simulations, a standard finite element mesh convergence study was conducted to ensure accuracy. This study can be found in a previous work [9].

## 3. Results and Discussion

In this text, the results and discussions are integrated into a cohesive section, facilitating a clear and coherent narrative. This approach prevents unnecessary repetition and strengthens the connection between findings and arguments, thereby simplifying the interpretation of outcomes.

**Figure 4:**
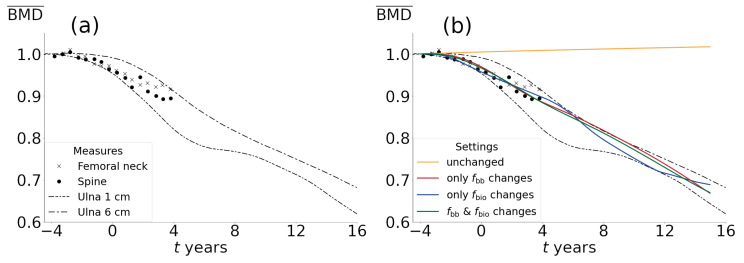
(**a**) Experimental measures of average bone mineral density relative change from the work by Recker et al. [40] (femoral neck and spine) and the work by Ahlborg et al. [41] (Ulna 1 cm and Ulna 6 cm). The measurements are expressed as relative values to the beginning of the perimenopausal stage. (**b**) Normalized space-averaged BMD ($\bar{\mathrm{BMD}}$) as a function of time for different fitting settings. The data can be fitted by different combinations of parameters. The resorption caused by increased bone turnover (model fitted solely on $f_{\mathrm{bio}}$) leads to subsequent bone formation, which can be seen in the undulations of the green curve.

The simulations cover up to 19 years of bone evolution (4 years prior and 15 years after the last menstrual period) allowing us to observe long-term trends. The goal is to establish a model that can effectively simulate the temporal evolution of bone mineral density in the entire femur model for women experiencing menopause, considering their engagement in normal or modified activities, and exploring combined situations involving both menopause and ageing. In this regard, there are two valuable experimental studies that we will use as benchmarks for comparing the outcomes of the current model. In the first work, Recker et al. [40] reported BMD measurements of the spine and femoral neck from almost five years before to almost five years after the last menses (9.5 years of measurements), collecting data from 75 women over 46 years old who had premenopausal estradiol and gonadotropin levels and regular menses. The results of the measurements are presented as relative BMD values with respect to the initial value before menopause ($\bar{\mathrm{BMD}}$). This work was used by Hernández et al. [22] to compare their computational results with experiments. For a longer period of measurements, Ahlborg et al. [41] evaluated bone loss in 156 women in the peri- and postmenopausal period, ranging in age from 48 to 64 years. All women were premenopausal at the beginning of the study. The areal bone mineral density ($\mathrm{mg}/{\mathrm{cm}}^{2}$) of the forearm was measured 1 cm and 6 cm proximal to the styloid process of the ulna every second year by single-photon absorptiometry. The average BMD value of the left and right forearms was used. For the present work, BMD values from the measurements by Ahlborg et al. [41] are converted to relative values compared to the initial ones, like those from Recker et al. [40], in order to include and compare trends of all experimental and computational results in each figure. The experimental data mentioned above are shown in Figure 4a.

Our analysis will first focus on fitting the model parameters ($\kappa_{\mathrm{perm}}$ and long-term trends for $f_{\mathrm{bio}}$ and $f_{\mathrm{bb}}$) to the experimental data, highlighting the effect of turnover frequency ($f_{\mathrm{bio}}$) and osteoblast/osteoclast balance (focal balance, $f_{\mathrm{bb}}$) variations on the tissue remodelling process. Then, we will examine the effects of overload and a sedentary lifestyle on $\bar{\mathrm{BMD}}$ evolution when compared to normal load.

### 3.1. Parameter Adjustment

According to the measurements by Recker et al. [40] and Ahlborg et al. [41], the relative BMD evolution (see Figure 4a) exhibits a significant decline during the perimenopausal period, followed by a sustained decrease due to ageing. This behavior aligns with the trends predicted by Hernández et al. [22] for a representative volume of trabecular bone when considering the simultaneous and permanent changes in the parameters that model the effects of bone turnover frequency ($f_{\mathrm{bio}}$) and focal balance ($f_{\mathrm{bb}}$). However, it is not entirely clear whether these effects alone can explain the mentioned changes and their respective influences on bone mass and mineral content, which can contribute to clarifying clinical interventions and/or training plans aimed at minimizing pathological effects. To analyze the individual effects of each parameter on bone changes, three scenarios are proposed: (1) variations only on $f_{\mathrm{bio}}$, (2) variations only on $f_{\mathrm{bb}}$, and (3) simultaneous variations in both parameters. A fourth theoretical scenario in which both parameters are unchanged is presented for comparison purposes. In the first three cases, the obtained curves correspond to those parameter values providing the best fit to the experimental data throughout the entire range by minimizing the mean squared error. The fitted parameter values are shown in Table 3. Figure 4b shows a good fit of these three cases during the perimenopausal period, capturing the evolution of $\bar{\mathrm{BMD}}$. In the postmenopausal period, however, although the decreasing trend is captured, there are important differences that will be discussed later. Indeed, certain aspects concerning the dynamics of each phenomenon reveal their relative biological plausibility. This leads us to explore fundamental aspects of the BMU complex’s mechanisms in order to gain new insights and identify clinically relevant factors. These aspects are discussed in more detail below.

The model calibration was carried out through a process of successive approximations. In the initial stage, simulations were performed using exploratory values for the parameters. From the obtained results, the curve of normalized bone mineral density over time was interpolated for intermediate parameter values. Subsequently, the mean squared error resulting from this interpolation was minimized, allowing for an approximation of the optimal parameters. Using these values as a starting point, additional simulations were conducted iteratively, aiming to progressively approach the optimal parameters in each iteration cycle.

### 3.2. Analysis in the Volume Fraction Space ($v_{b}$): The Effects Solely Caused by Variations in $f_{\mathrm{bio}}$ Are Biologically Unlikely

**Figure 5:**
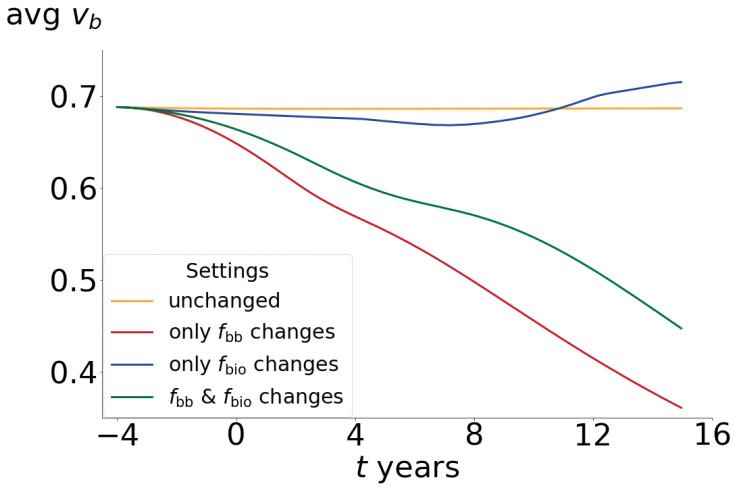
Space-averaged $v_{b}$ as a function of time for different fitting settings. The increase in bone turnover does not lead to a net imbalance, and therefore, the volume fraction does not significantly change. Instead, this results in a decrease in mineral content and a net formation of bone at the end of the simulated period due to the mechanical stimulation caused by its weakening (green curve). The focal balance drift towards resorption implies a significant decrease in bone volume fraction (red curve).

In the case of changes solely driven by $f_{\mathrm{bio}}$, where the focal balance remains unchanged, a tissue with a higher proportion of osteoid but not a significant change in bone volume would be expected. Figure 5 shows that the average volume fraction ($v_{b}$) throughout the entire femur, representing the average amount of bone, does not undergo significant changes, despite the decrease in $\bar{\mathrm{BMD}}$ observed in Figure 4b. These findings carry implications for predictions during the postmenopausal period, indicating that changes solely attributed to $f_{\mathrm{bio}}$ predict smaller losses in comparison to the other two scenarios.

The reason for this is that increased BMU activity would lead them to replace more internally located, and therefore more mineralized, bone segments [8] because the new and superficial ones do not have enough time to mineralize and be replaced again. Despite this, as the overall amount of bone remains relatively stable, there are newly formed bone segments that mineralize at a slower rate, leading to slightly higher BMD values at the end of the simulation period compared to the other scenarios. If this phenomenon was actually possible in reality, it would result in bone with increased osteoid content and decreased mineral content but with a mostly constant amount of bone, which is unlikely to occur as menopause and ageing are strongly associated with significant bone loss [42]. Furthermore, Table 4 illustrates the percentage increase in porosity measurements of the tibia and radius bones extracted from work by Bjørnerem et al. [42], compared to the average values of the simulations. It is evident that, in the case of changes solely driven by $f_{\mathrm{bio}}$, the predicted bone volume loss by the model is very small, while experimental measurements show significant changes (2.43%±0.15% change on total cortical porosity for tibia at peri- to postmenopause transition vs. 0.4% on simulation results). Thus, it is highly unlikely for BMD reduction to occur solely due to increased bone turnover, as its effect does not align with the data indicating increased porosity. Indeed, a long-term recovery in the volume fraction can be observed (see Figure 5). This is because the decrease in the average elastic modulus (due to a reduction in the ash fraction) is sufficient for the mechanical stimulus to enter the bone formation zone until the accommodation process leads it to the dead zone. Since this mechanism remains unaltered, the amount of bone remains almost stable. It is clear that the factors that affect focal balance (changes in $f_{\mathrm{bb}}$) appear to play a significant role in the process of BMD and bone mass loss. However, the predictions of increased porosity solely due to variations in this parameter result in much higher average losses compared to the maximum observed during each period (2.43%±0.15% change in total cortical porosity for tibia at peri- to postmenopause transition vs. 5.23% on simulation results). It is worth noting that the experimental data used to adjust the parameters and those presented in Table 4 [42] are from different bones. Although bone tissue behavior is generally independent of its location, it is highly dependent on its initial state and everyday use. Therefore, these results should be considered in terms of general trends and approximate value ranges. In this regard, the combined effect of both parameters yields porosity percentage increase values that are more consistent with the experimental data in terms of bone loss percentage. While the curve obtained by varying only $f_{\mathrm{bb}}$ can excellently fit the BMD evolution, disregarding the fact that it shows significantly higher values of bone mass loss (porosity) compared to the experimental data, there are factors related to bone density evolution that would indicate that this case may not be feasible. These factors will be discussed below.

### 3.3. Analysis in the Density Space: Resorption Solely Caused by Increased $f_{\mathrm{bb}}$ Has Improbable Effects in Practice

**Figure 6:**
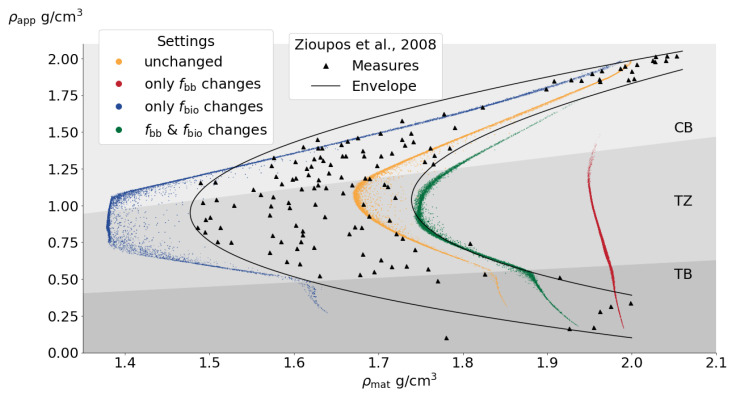
$\rho_{\mathrm{app}}$ vs. $\rho_{\mathrm{mat}}$ at 15 years after last menses. Experimental data from the work by Zioupos et al. [30]. Different shaded areas show the regions of cortical bone (CB), trabecular bone (TB) and transition zone (TZ) [8]. Every point on the graph represents the value of $\rho_{\mathrm{mat}}$ and $\rho_{\mathrm{app}}$ at a specific location within the modeled femur. The points are distributed to cover all internal regions of the femur model.

**Figure 7:**
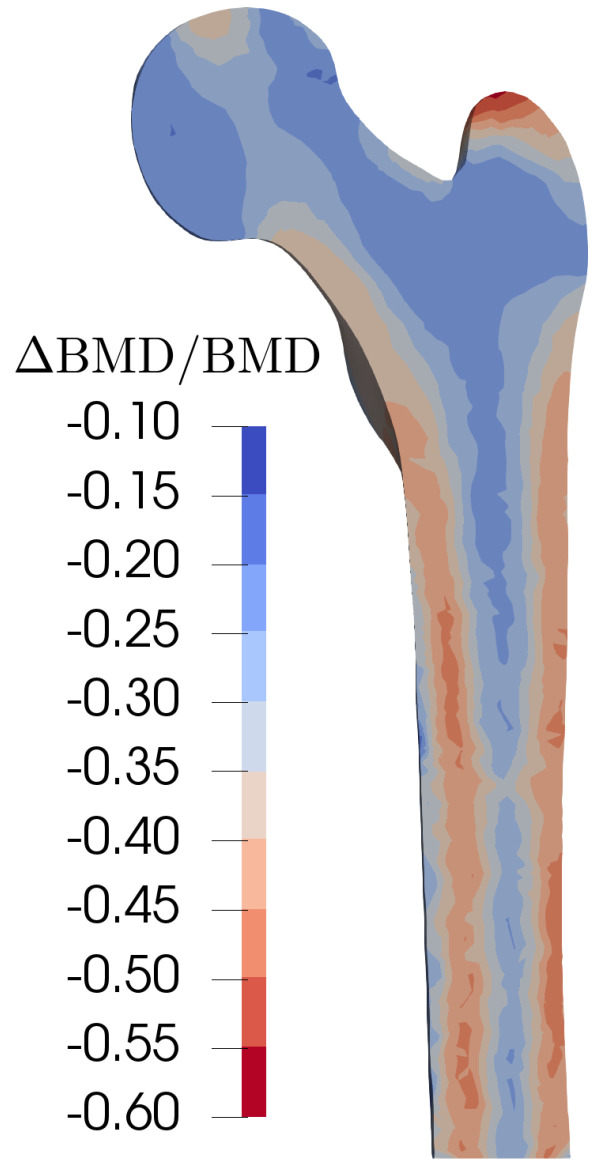
Relative change in BMD (simulated) at 15 years from last menses due to simultaneous increases in bone turnover ($f_{\mathrm{bio}}$) and decreases in focal bone balance ($f_{\mathrm{bb}}$) leading to resorption.

In this case, we will examine the results of the mentioned cases by comparing the model data with the experimental measurements of the $\rho_{\mathrm{mat}}$—$\rho_{\mathrm{app}}$ relationship obtained from the work by Zioupos et al. [30], which were previously used to validate the results of the previous version of the current model [8,9]. The individual scenarios of (1) increased bone turnover and (2) increased focal balance have opposing effects. When resorption is driven by $f_{\mathrm{bb}}$, the boomerang-like curve shifts to the right (Figure 6), indicating that, in general, bone fragments are more mineralized (resulting from increased $\rho_{\mathrm{mat}}$). This phenomenon arises due to net resorption, wherein the less mineralized superficial bone fragments gradually diminish over time [8]. Conversely, the more internal fragments, characterized by higher mineral content per unit volume, are eventually the last to be resorbed. Consequently, this leads to a more fragile tissue [43]. However, it should be noted that the most significant effect in this case is the drastic reduction in cortical bone, according to the division of previous studies [8], causing the cortical zone to virtually disappear throughout the bone (see Figure 6). This phenomenon can be attributed to net resorption, which increases the porosity of the cortical zone, thereby expanding its free surface area (see Figure 7) and enhancing the activity of the BMUs. To the best of the authors’ knowledge, it is highly improbable for the compact bone to completely disappear in reality. On the other hand, when resorption is driven by $f_{\mathrm{bio}}$, the curve shifts dramatically to the left, indicating a higher proportion of osteoid content and a lower mineral content (resulting from a reduction in material density), which further supports the previously discussed results in the volume fraction space. Together, the results suggest that adjusting the BMD curves by independently varying each parameter ($f_{\mathrm{bio}}$ and $f_{\mathrm{bb}}$) leads to unrealistic outcomes. Therefore, a consistent model would be expected to simultaneously involve changes in $f_{\mathrm{bio}}$ and $f_{\mathrm{bb}}$ in order to encompass both hormonal and ageing-related remodeling changes.

### 3.4. Simultaneous Changes in f*_bio_* and f*_bb_* Lead to Both Realistic Bone Loss over Time and Mineralization Distribution

In the previous sections, we showed that a combined change in the parameters $f_{\mathrm{bio}}$ and $f_{\mathrm{bb}}$ is necessary for the prediction of BMD evolution to be biologically plausible. In this case, Figure 4b demonstrates good agreement between the numerical results and experimental data for normal activities, i.e., maintaining pre-menopausal loads. It can be observed that tissue losses fall within reasonable values compared to the data in Table 4. Simulation results showed changes in porosity at different stages: 1.89%, 4.01%, and 3.14% from pre- to perimenopause, peri- to postmenopause, and postmenopause, respectively. These values are similar to experimental measurements of changes in compact bone porosity in tibia, which were 1.81% ± 0.19%, 3.64% ± 0.34%, and 2.00% ± 0.15% for comparable stages. The density ratio (Figure 6) exhibits two aspects: on the one hand, there is an increase in mineral content per unit volume due to net resorption (effects of $f_{\mathrm{bb}}$), which leads to a more fragile tissue [43]. On the other hand, a reduction in tissue is also noticeable in the cortical zone, although to a lesser extent than when $f_{\mathrm{bb}}$ alone varies. It is interesting to note that the areas of maximum BMD loss are found in the most structural part of the bone. Figure 7 shows the areas of greatest BMD loss in red and the areas of least BMD loss in blue. The losses are computed by comparing the final state of the bone (19 years after menopause beginning) with the initial bone, prior to the onset of menopause. The complete femur model allows for visualization of the maximum losses concentrated in the areas of compact tissue, precisely the tissue that supports the maximum loads of flexion and torsion. It is evident that the structural integrity of the femur, particularly in the diaphysis, deteriorates, which can increase the risk of fractures in older individuals. The BMU activity is enhanced due to the amount of free surface growth (increase of porosity), which enters into negative feedback for the cortical zone and adds to the increased loss due to the changes in $f_{\mathrm{bio}}$ and $f_{\mathrm{bb}}$. Conversely, although there is a reduction in BMD in the more spongy and internal areas of the bone, they are already at one extreme of the available surface/porosity curve, so there is no growth in activity beyond that implied by menopause and ageing factors. The relationship between free bone surface and porosity harms the more compact areas and, therefore, their structural integrity and mechanical capacity once osteoporosis is reached. This is one of the reasons why some inevitable losses in the ability to bear loads occur and the reason why special attention should be paid to bone growth and strengthening in the years leading up to menopause, as was pointed out by Hernández et al. [21]. However, once menopause is reached, a question arises regarding the natural improvement or deterioration in bone health over time. In line with this objective, we will now delve into the potential impact of increasing or decreasing physical activity to examine how modifying loads can affect long-term bone health once prevention is no longer an option.

### 3.5. Exercise during the Perimenopause Stage Delays the Onset of Osteoporosis

To assess the evolution of BMD under modified loads, the osteoporosis threshold was defined in terms of $\bar{\mathrm{BMD}}$. Osteoporosis is diagnosed when the BMD of a specific bone region falls below 2.5 standard deviations from the healthy value of young women [29]. The osteoporosis threshold was calculated using total femur BMD female data from Looker et al. [44] and the following equation:

(16) $${\bar{\mathrm{BMD}}}_{\mathrm{th}}=\frac{{\mathrm{BMD}}_{s}-{\mathrm{BMD}}_{\mathrm{SD}}}{{\mathrm{BMD}}_{\mathrm{ref}}}$$

In this equation, BMDs represents the healthy value, which is the average between 20- and 29-year-old women ($N_{s}=1380$), ${\mathrm{BMD}}_{\mathrm{SD}}$ is the standard deviation between them, and ${\mathrm{BMD}}_{\mathrm{ref}}$ is the average value between the women of ages from 40 to 59 ($N_{\mathrm{ref}}=2033$). From the tables provided by Looker et al. [44], the osteoporosis threshold was calculated for the total femur (see Table 5). Depending on the population, this threshold has some variations—0.720 for non-Hispanic white ($N_{s}=409$, $N_{\mathrm{ref}}=894$), 0.698 for non-Hispanic black ($N_{s}=492$, $N_{\mathrm{ref}}=645$), and 0.727 for Mexican white ($N_{s}=479$, $N_{\mathrm{ref}}=494$), but all are inside the interval of 0.70–0.73, and these little variations do not affect the conclusion. A value of 0.72 will be used to compare different load scenarios.

By using the model parameters for combined changes, in this section we will compare the $\bar{\mathrm{BMD}}$ evolution for a normal load and two opposite situations: a moderate increase of 30% in load to simulate sustained physical activity (overload), and a reduction of the same percentage to simulate a trend towards sedentary behavior.

**Figure 8:**
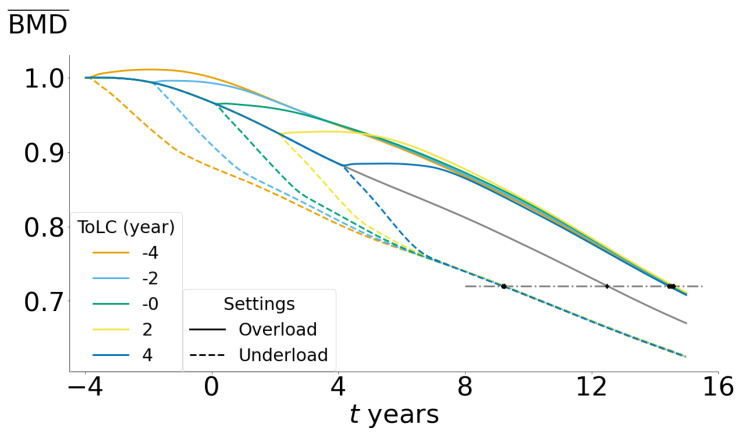
Normalized space-averaged BMD ($\bar{\mathrm{BMD}}$) as a function of time for overload and underload settings. ToLC: time of load change. Overload: 30% load increase. Underload: 30% load decrease. Osteoporosis threshold ($\bar{\mathrm{BMD}}$ = 0.7) represented by dash-dotted horizontal line.

In the first scenario, represented by Figure 8, it is evident that, regardless of the time of the initiation of physical activity, there is a consistent increase in $\bar{\mathrm{BMD}}$ compared to normal activity. Furthermore, this increase is sustained even after menopause, as long as activity remains. In all cases, there is a temporary boost in $\bar{\mathrm{BMD}}$ while the bone re-adapts to the new situation, followed by a subsequent evolution of $\bar{\mathrm{BMD}}$ along a curve that closely parallels the original curve. On the other hand, a decrease in activity leads to a pronounced drop of $\bar{\mathrm{BMD}}$, stabilizing the slope over time and resulting in a net bone change over time that is similar to the normal case. This behaviour is closely related to the parameter ϕ that accounts for the tissue’s adaptive capacity to external stimuli. The ϕ value used in this study demonstrated highly accurate predictions of bone mass evolution in response to disuse in a previous work [9].

These findings have an important implication: it is never too late to engage in physical activity, as this has the potential to mitigate the deterioration in the material composition of bone tissue. This effect is particularly relevant before and during menopause, emphasising the importance of maintaining consistent and long-term engagement in physical activity. On the contrary, sedentary behaviour, which is commonly observed in older women, accelerates the decline in BMD, posing a significant risk of early osteoporosis, which increases the likelihood of fractures. It is worth noting that the effects of overload/underload regimes are not exactly mirrored, as the effects of disuse are more harmful than their overuse counterpart benefits. In fact, our results suggest that a moderate decrease in mechanical stimulation can precipitate the onset of osteoporosis by up to 6 years when compared to bones that receive adequate stimulation. Our findings also highlight the potential for additional improvement by implementing higher levels of controlled stimulation, which were not within the scope of this study.

### 3.6. Limitations

While our model holds significant promise for future research, it is important to acknowledge certain limitations. First, it is based on a continuum approach, which is not suitable for investigating the effects of trabecular thinning. Additionally, it cannot predict the impact of connectivity loss due to trabecular removal, which can affect strain distribution and, consequently, local remodelling. Furthermore, the model does not currently account for the effect of microdamage, which limits its ability to predict the maximum loads that can be applied, before or after the onset of osteoporosis, without causing local failures. This, however, can and will be incorporated in future versions of the model.

## 4. Conclusions

This work introduces a bone remodelling model governed by mechanical loads, incorporating the effects of osteoporosis and ageing through constant variation functions that weigh the activity of BMUs (frequency of one turnover) and focal balance. In contrast to previous works, this model assumes only constant (irreversible) variations in these factors, which persist and continue to vary even after menopause to account for ageing. These functions are adjusted so that the model’s outcomes under normal loads align with the experimental data.

According to the results of this work, the effects of increased bone turnover (frequency of BMU activation, $f_{\mathrm{bio}}$) and net removal (reduced focal balance, $f_{\mathrm{bb}}$) must be simultaneously present, with a constant variation over time. These effects lead to a consistent and inevitable loss of BMD and bone mass with age, resulting in age-related osteoporosis that depends on a mechanical stimulation of the bone, among other factors. It should be noted that while $f_{\mathrm{bio}}$ does not directly affect mass, its increase enhances the frequency of BMU activation with reduced $f_{\mathrm{bb}}$ (focal balance). Controlling $f_{\mathrm{bio}}$ will impact the control of both factors simultaneously, while controlling only $f_{\mathrm{bb}}$ would reduce bone mass losses to a greater extent. These effects can be achieved by planning training programs that gradually increase physical activity. The transient effect of medications can easily be incorporated into the model by adjusting the shape of the κ(t) curves. The calibration of the model for these scenarios is beyond the scope of this paper.

By emphasizing the significance of maintaining an active lifestyle, especially during menopause and as individuals age, they can proactively safeguard their bone health. The study’s crucial findings demonstrate that engaging in moderate physical activities can delay the onset of osteoporosis by up to 6 years when compared to a sedentary lifestyle, thus reducing the likelihood of developing severe osteoporosis in old age. Moreover, the potential for a greater reduction exists if physical activity programs are implemented to increase activity and further delay or even prevent the onset of osteoporosis.

Overall, the model exhibited an excellent ability to fit the experimental data in previous studies, and an exceptional ability to adapt to the BMD evolution in this study. Once the model was aligned with experimental measurements, it showed excellent predictive capabilities for the average bone density evolution under both normal and pathological conditions. In this sense, the results of this study are promising not only for investigations into the interplay between hormonal factors and BMU activity, but also for predicting the potential effects on the natural prevention of osteoporosis through the personalized planning of physical activities. Subsequent studies with our model will explore the impact of targeted hormonal treatments in conjunction with planned training.

## Figures and Tables

**Table 1:** Bone remodeling model parameters. Based on previous works [8,9].

Parameter	Description	Value
Tissue composition		
$\rho_{m}$	Mineral density	3.2 g/cm^3^
$\rho_{o}$	Organic density	1.1 g/cm^3^
$\rho_{w}$	Water density	1.0 g/cm^3^
$v_{o}$	Organic volume fraction	3/7
Remodeling		
$T_{R}$	Resorption period	24 days
$T_{I}$	Reversal period	18 days
$T_{F}$	Formation period	64 days
$v_{\mathrm{UBM}}$	BMUs speed	0.04 mm/day
*m*	Load—cycles weight parameter	4
$f_{\mathrm{bb}}^{\min}$	Min. pre-κ $f_{\mathrm{bb}}$	0.85
$f_{\mathrm{bb}}^{\max}$	Max. pre-κ $f_{\mathrm{bb}}$	1.15
*−w*	$f_{\mathrm{bb}}$ dead zone width	1.5 × 10^−3^
*−v*	$f_{\mathrm{bb}}$ linear zone width	1.5 × 10^−3^
ϕ	Adaptation rate	1.0 × 10^−4^
$f_{\mathrm{bio}}$	Max. activation frequency	5.0 × 10^−3^

**Table 2:** Applied loads. Walking loads at maximum contact force. Stair-climbing loads at maximum torsional moment. Location are depicted in Figure 3. Based on work by Heller et al. [39].

Pattern	Load (N)	$F_{x}$	$F_{y}$	$F_{z}$	Location
Walking	Hip contact	−459.0	−278.8	−1948.2	$P_{0}$
	Abductor	493.0	36.6	735.3	$P_{1}$
	Tensor fascia latae, proximal	61.2	98.6	112.2	$P_{1}$
	Tensor fascia latae, distal	−4.3	−6.0	−161.5	$P_{1}$
	Vastus lateralis	−7.7	157.3	−789.7	$P_{2}$
Stair-climbing	Hip contact	−504.1	−515.1	−2008.6	$P_{0}$
	Abductor	595.9	244.8	721.7	$P_{1}$
	Iliotibial band, proximal	89.3	−25.5	108.8	$P_{1}$
	Iliotibial band, distal	−4.3	−6.8	−142.8	$P_{1}$
	Tensor fascia latae, proximal	26.4	41.7	24.7	$P_{1}$
	Tensor fascia latae, distal	−1.7	−2.6	−55.3	$P_{1}$
	Vastus lateralis	−18.7	190.4	−1148.4	$P_{2}$
	Vastus medialis	−74.8	336.6	−2270.4	$P_{3}$

**Table 3:** Fitted parameter values for the menopause and ageing model. The κ parameters correspond to those shown in Figure 2a.

Parameter	$f_{\mathrm{bio}}$	$f_{\mathrm{bb}}$
Fitted solely on $f_{\mathrm{bio}}$		
$\kappa_{\mathrm{perm}}$	1.489	1.000
long term $\frac{d\kappa }{dt}$	3.654 × 10^−4^	0.000
Fitted solely on $f_{\mathrm{bb}}$		
$\kappa_{\mathrm{perm}}$	1.000	0.793
long term $\frac{d\kappa }{dt}$	0.000	−7.085 × 10^−5^
Fitted on both $f_{\mathrm{bio}}$ and $f_{\mathrm{bb}}$		
$\kappa_{\mathrm{perm}}$	1.230	0.903
long term $\frac{d\kappa }{dt}$	1.610 × 10^−4^	−4.452 × 10^−5^

**Table 4:** Average annual percentage (%) increase of porosity for different stages of menopause. The upper rows display the experimental data for the distal region of the tibia and the distal region of the radius, measured in the cortical zone and in the external and internal transitional zone to trabecular bone. The three lower rows show the annual percentage increases in porosity extracted from the current model, averaged throughout the simulated bone. Experimental data from the work by Bjørnerem et al. [42]. CB: compact bone; TZ: transitional zone.

Experimental Measures			
	**Pre- to Peri-**	**Peri- to Post-**	**Postmenopause**
Age (years), mean (range)	47.5 (40–54)	51.8 (46–55)	60.5 (48–75)
Tibia, distal region			
Sample size, *n*	56	34	118
Δ Total cortical porosity (%)	1.43±0.11	2.43±0.15	1.35±0.10
Δ Cortical porosity, CB (%)	1.81±0.19	3.64±0.34	2.00±0.15
Δ External TZ porosity (%)	1.56±0.16	3.19±0.27	1.98±0.15
Δ Internal TZ porosity (%)	0.44±0.03	0.63±0.04	0.36±0.03
Radius, distal region			
Sample size, *n*	43	24	90
Δ Total cortical porosity (%)	1.22±0.16	2.31±0.22	1.53±0.13
Δ Cortical porosity, CB (%)	1.84±0.25	3.78±0.45	2.18±0.18
Δ External TZ porosity (%)	1.45±0.19	3.23±0.40	1.95±0.16
Δ Internal TZ porosity (%)	0.22±0.03	0.40±0.06	0.26±0.03
Simulations			
Relative age to last menses (years)	−4 to 0	0 to 4	4 to 15
Fitting settings	Δ Total porosity (%)		
only $f_{\mathrm{bio}}$ changes	0.58	0.40	−1.18
only $f_{\mathrm{bb}}$ changes	3.03	5.23	3.65
$f_{\mathrm{bio}}$ and $f_{\mathrm{bb}}$ changes	1.89	4.01	3.14

**Table 5:** Osteoporosis threshold in terms of normalized bone mineral density (${\bar{\mathrm{BMD}}}_{\mathrm{th}}$). Computation of threshold values is based on the work by Miller [29]. Total femur BMD data from female subjects from the work by Looker et al. [44].

Variable	Age Range (Years)	Non-Hispanic White (Sample Size)	Non-Hispanic Black (Sample Size)	Mexican White (Sample Size)
${\mathrm{BMD}}_{s}$ $\left(g/{\mathrm{cm}}^{2}\right)$	20–29	0.942 (409)	1.026 (492)	0.950 (479)
${\mathrm{BMD}}_{\mathrm{SD}}$ $\left(g/{\mathrm{cm}}^{2}\right)$	20–29	0.122 (409)	0.134 (492)	0.113 (479)
${\mathrm{BMD}}_{\mathrm{ref}}$ $\left(g/{\mathrm{cm}}^{2}\right)$	40–59	0.885 (894)	0.990 (645)	0.919 (494)
${\bar{\mathrm{BMD}}}_{\mathrm{th}}$ (Equation (16))		0.720	0.698	0.727

## Data Availability

The data presented in this study are available on request from the corresponding author.
